# Smoking among morbidly obese patients

**DOI:** 10.1186/1471-2466-10-61

**Published:** 2010-11-24

**Authors:** Raquel Chatkin, Claudio C Mottin, José M Chatkin

**Affiliations:** 1 Nutritionist, Morbid Obesity Center at the Hospital São Lucas da Pontifícia Universidade Católica do Rio Grande do Sul (PUCRS), Porto Alegre, Brazil; 2 Professor, Post-Graduation Program in Medicine and Health Sciences, School of Medicine, PUCRS; Morbid Obesity Center at the Hospital São Lucas da PUCRS. Porto Alegre, Brazil; 3 Professor, Post-Graduation Program in Medicine and Health Sciences, School of Medicine, PUCRS, Porto Alegre, Brazil

## Abstract

**Background:**

Smokers usually have a lower Body Mass Index (BMI) when compared to non-smokers. Such a relationship, however, has not been fully studied in obese and morbidly obese patients. The objective of this study was to evaluate the relationship between smoking and BMI among obese and morbidly obese subjects.

**Methods:**

In a case-control study design, 1022 individuals of both genders, 18-65 years of age, were recruited and grouped according to their smoking status (smokers, ex-smokers, and non-smokers) and nutritional state according to BMI (normal weight, overweight, obese, and morbidly obese).

**Results:**

No significant differences were detected in the four BMI groups with respect to smoking status. However, there was a trend towards a higher frequency of smokers among the overweight, obese, and morbidly obese subjects compared to normal weight individuals (p = 0.078). In a logistic regression, after adjusting for potential confounders, morbidly obese subjects had an adjusted OR of 2.25 (95% CI, 1.52-3.34; p < 0.001) to be a smoker when compared to normal weight individuals.

**Discussion:**

In this sample, while the frequency of smokers diminished in normal weight subjects as the BMI increased, such a trend was reversed in overweight, obese, and morbidly obese patients. In the latter group, the prevalence of smokers was significantly higher compared to the other groups. A patient with morbid obesity had a 2-fold increased risk of becoming a smoker. We speculate that these finding could be a consequence of various overlapping risk behaviors because these patients also are generally less physically active and prefer a less healthy diet, in addition to having a greater alcohol intake in relation to their counterparts. The external validity of these findings must be confirmed.

## Background

The interrelationship between smoking and body weight is already known based on several studies most of them published in the 1990s [1,2].

Smokers tend to have a lower Body Mass Index (BMI), when compared to non-smokers, paired by gender and age. Large cross-sectional studies observed a significant inverse relationship between regular tobacco use and body weight, and such a relationship tends to be lower among smokers compared to non-smokers [3-5]

The relationship between smoking and BMI was reviewed by Chiolero et al. [6]. They analyzed studies conducted in the 1980s which showed that smoking a cigarette induces a 3% increase in energy expenditure within a few minutes. By smoking 24 cigarettes in 1 day, the increase in total energy expenditure can be > 2200 kcal. In contrast, heavy smokers tend to have a greater BMI, probably as a consequence of several risky behaviors, such as poor diet, low physical activity, and alcohol use.

Smoking and obesity are independent health risk factors. Koster et al. [7] described the interrelationship between BMI, smoking, and mortality. They reported an increased risk of mortality among subjects with a BMI > 35 kg/m^2^. They also described, after adjustment for BMI and other covariates, a higher risk of mortality in former and current smokers of both genders. Peto et al. [8] recently reported the effects of obesity and smoking on life expectancy.

The very frequent co-existence of these two clinical situations (smoking and adiposity) needs to be better understood [9]. The aim of this study was to evaluate the frequency of these two health problems in a population seeking medical assistance at a clinic for morbidly obese subjects.

## Methods

### Participants and sampling

In a case-control study, we collected data from patients who attended the Morbid Obesity Center at the Hospital São Lucas Pontificia Universidade Catolica do Rio Grande do Sul (PUCRS) (source of cases) and blood donors of the same hospital (source of controls). Data were collected between January 2006 and December 2007.

The inclusion criteria were as follows: both genders; 18-65 years of age; smokers, non-smokers, and ex-smokers; and a BMI ≥ 18.5 kg/m^2^. The exclusion criteria were the presence of any psychopathies, abusive use of legal (alcohol) or illegal drugs, and pregnant women or women who were breastfeeding. In addition to these criteria, the volunteers of the control group underwent prior screening in the Blood Center of the Hospital.

The volunteers were grouped according to smoking status. Current smokers were those who had smoked ≥ 100 cigarettes in their lifetime and were still smoking daily or smoking on most days. Non-smokers were those who had never smoked or smoked < 100 cigarettes in their lifetime and currently were not smoking. Ex-smokers were those who smoked > 100 cigarettes in their lifetime, but had not smoked for > 90 days. This latter criteria is used to allow a patient to be submitted to a bariatric surgey.

The patients were classified based on their BMI, in accordance with the World Health Organization (WHO) criteria [10] as individuals of normal weight (BMI = 18.5-24.9 kg/m^2^), overweight (BMI = 25.0-29.9 kg/m^2^), obese (BMI = 30-34.9 kg/m^2^), and morbidly obese (BMI ≥ 35 kg/m^2^).

The studied variables included height, weight, BMI, gender, age, level of education, and smoking status (non-smoker, smoker, or ex-smoker).

### Statistical analysis

The continuous data were described by the mean and standard deviation. In the presence of asymmetry and for some select variables, the median, interquartile range, and total range were used. The categorical variables were described by counts and percentages. Comparisons of groups were done using analysis of variance for the continuous data, as well as the chi-square test for categorical data.

The association between the categorized BMI and smoking status was evaluated in a logistic regression model including the following factors: age; gender; and level of education. The level of significance was set at an α = 0.05. The data were analyzed with SPSS (version 17.0).

The present work did not pose any risk of death or contamination to the patients. The secrecy of the information was guaranteed. The study was approved by the Scientific Commission and the Committee of Ethics in Research of Hospital São Lucas Pontificia Universidade Catolica do Rio Grande do Sul.

## Results

The profiles of the 1022 individuals included in the study are shown in Table 1, where the data are stratified according to BMI (normal weight, n = 353, overweight n = 283; obese n = 82; and morbidly obese n = 304). Normal weight individuals had a lower mean age when compared to the other subgroups (p < 0.001). The frequency of women among the morbidly obese was higher in relation to other groups (p < 0.001). Individuals with > 12 years of schooling had a higher frequency of morbid obesity (p < 0.001).

**Table 1:** Characteristics of the individuals according to BMI

Variable	Total n = 1022	Normal weight N = 353	Overweight n = 283	Obese n = 82	Morbidly obese n = 304	P
Age, years	38.3 ± 11.1	35.0 ± 10.4	40.5 ± 11.22	39.4 ± 10.5	39.9 ± 11.3	< 0.001
Males, No. (%)	524(51.38)	196 (55.8)	183 (65.1)	51 (62.2)	94 (30.9)	< 0.001
Education level, years						< 0.001
Up to 8 (%)	403 (39.4)	139 (39.4)	161 (56.9)	49 (59.8)	33 (10.8)	
9 to 12 (%)	384 (37.5)	140 (39.7)	90 (31.8)	22 (26.8)	138 (45.3)	
> 12 (%)	235 (22.9)	74 (21.0)	32 (11.3)	11 (13.4)	133 (43.7)	
BMI, kg/m^2^						< 0.001
Mean	31.9 ± 11.427.0	22.6 ± 1.5	27.0 ± 1.3	31.9 ± 1.4	47.2 ± 8.7	
Median		22.8	26.8	31.6	45.3	
Min and Max	19.9 - 92.2	17.9 - 25.0	25.0 - 29.89	30.1 - 34.8	35.1 - 92.2	
Weight, kg						< 0.001
Mean	89.9 ± 32.9	65.2 ± 7.8.1	76.5 ± 9.2	90.5 ± 8.8	131.0 ± 30.1	
Median	79	64.0	76.0	89.5	124.0	
Min and Max	47-242.0	47.0-90.0	56.0-105.0	72.0-113.0	79.0-242.0	
Smoking habit, No. (%)						0.078
Non-smokers	568 (55.6)	207 (58.5)	155 (54.8)	43 (52.4)	164 (53.8)	
Smokers	265 (25.9)	84 (23.9)	63 (22.3)	24 (29.3)	94 (31.0)	
Ex-smokers	189 (18.4)	62 (17.6)	65 (23.0)	15 (18.3)	46 (15.2)	

The data are presented as the means ± standard deviation, number (percent), or median (P25-P75).

P: statistical significance with Student's t-test and Pearson's chi-squared test.

The mean BMI values were 22.6 ± 1.5 kg/m^2^, 27.0 ± 1.3 kg/m^2^, 31.9 ± 1.4 kg/m^2^, and 47.2 ± 8.7 kg/m2 for normal weight, overweight, obese, and morbidly obese patients, respectively (p < 0.001).

There was no statistically significant difference among the BMI groups with respect to smoking status. However, a trend for a greater number of smokers was noted in the morbidly obese group when compared to the other groups, albeit without statistical significance (p = 0.078).

**Figure 1:**
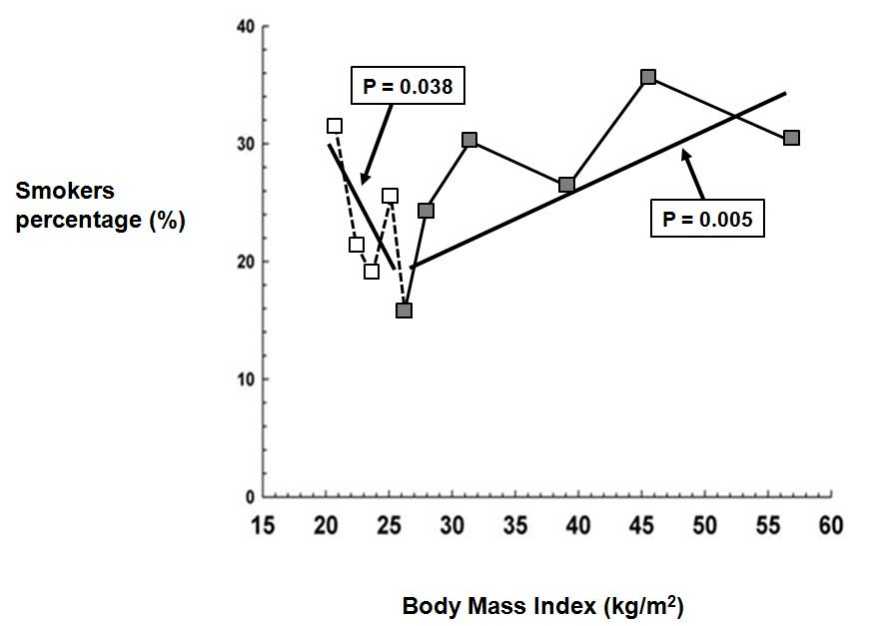
**Line graph representing the relationship between body mass index and percentage of smokers**. Normal weight: dotted lines; overweight, obese and morbidly obese: continuous line

Figure 1 shows the percentage of smokers according to the BMI when considered as a continuous variable. Among the individuals of normal weight (dotted line), there was a decline in the prevalence of smokers as the BMI increased (represented as a thick continuous line; p = 0.038). This trend began in the overweight group and continued among the obese and morbidly obese individuals, in which the percentage of smokers increased as the BMI increases (represented as the second part of the thick continuous line; p = 0.005).

Table 2 presents the odds ratios (OR) and the 95% confidence intervals (95% CIs) categorized according to the BMI groups, after controlling for gender, age and education in a logistic regression model. The OR for being a smoker in the morbidly obese group was 2.25 (95% CI, 1.52-3.34; p < 0.001). The OR for being a smoker in the overweight and obese groups was 0.83 (95% CI, 0.57-1.21; p = 0.33) and 1.18 (95% CI, 0.68-2.03; p = 0.56), respectively. We also tested for interaction terms between BMI and gender (p = 0.38), age (p = 0.68), education (p = 0.94), and found no significant effects. Additionally, we did not find a non-linear effect of age (p = 0.98).

**Table 2:** Odds ratio for smoking according to BMI

Variable	%	OR*	95% CI	P
Normal weight		1	─	< 0.001
Overweight	22.3	0.80	0.55-1.18	0.26
Obese	29.3	1.16	0.67-1.99	0.60
Morbidly obese	31.0	2.18	1.46-3.25	< 0.001

*Odds ratio obtained in a logistic regression model including age, gender, and education level.

## Discussion

In this report, the relationship between smoking and BMI was confirmed. What this study adds to the existing literature is that in the group of individuals with morbid obesity the percentage of smokers increased as the BMI increased.

The group of volunteers with BMI ≤ 24.9 kg/m^2 ^had a smoking habit in agreement with what is known; specifically, smoking is associated with a lower body weight [11]. In our sample, as weight or BMI increased when analyzed as a continuous variable, there was a corresponding decrease in the percentage of smokers up to the BMI cut-off point. However, in overweight, obese, or morbidly obese smokers, the trends were different. As the BMI surpassed 25 kg/m^2^, the cut-off point for excessive weight, and was also analyzed as a continuous variable, the percentage of smokers among the groups began to increase, reaching a greater frequency in the morbidly obese, even after adjusting for various factors. Thus, the higher the BMI, the higher the percentage of smokers detected (p = 0.005).

Using logistic regression adjusting for confounders and categorizing the individuals into the 4 BMI groups previously described, the adjusted OR for morbidly obese subjects to be smokers was 2.25 (95% CI, 1.52-3.34; p = 0.001) compared to subjects of normal weight. In the other categories, even after adjustments, no statistical significance was demonstrated.

These findings lead to the supposition that in our sample, the well-known inverse relationship between BMI/body weight and smoking is valid only for persons with a BMI ≤ 25 kg/m^2^. Among the overweight, obese, and morbidly obese volunteers, the findings suggest an increased frequency of smokers, linear with the increase in BMI/body weight.

This relationship between BMI and smoking describes an U shape curve, similar to association between alcohol use and cardiovascular disease.

Our results are in agreement with the data reported by Chiolero et al. [12], in which a change in the frequency of smoking behavior among the obese compared to the normal weight individuals was noted; however, the morbidly obese individuals were not studied.

Chiolero et al. [6] speculated that the association between smoking and obesity may be due to reverse causality. Thus, overweight or obese people (and perhaps the morbidly obese) are more prone to be smokers and smoke higher quantities with the mythical goal of trying to lose weight. One can also speculate that this could be a consequence of overlapping of various risk behaviors because these patients also are generally less physically active and prefer a less healthy diet, in addition to having a greater alcohol intake in relation to their counterparts [13,14] Also, it is possible that higher stress and psychological disturbances may contribute to the need of smoking. Numerous reports indicate that not only does the group of morbidly obese individuals show a compulsion for unhealthy food and smoking, but also for alcohol, gambling, and other addictions [6,13].

Some authors [12,15] have reported that the risk of being obese increased with the number of cigarettes smoked per day. Smokers with a greater smoking load also showed a higher BMI in a report by Nielsen et al. [16] In the ATTEMPT study, a multinational cohort project that studied 2009 smokers at baseline, a higher BMI was associated with a higher consumption of cigarettes [17]; the subjects with a BMI > 27 kg/m^2 ^had a high association of being a smoker of > 20 cigarettes/day. Thus, there are various facts indicating that smoking is associated with a higher BMI in a dose-dependent manner, resulting in an increase in the prevalence of obesity in individuals with a higher smoking load.

However, Koster et al. (7) in 2008 [7] and 2009 [18] published that the percentage of smokers decreased as the BMI increased, even among subjects with a BMI > 35 kg/m^2^. The explanation of these opposing results could be that different factors were or were not included in the adjustment model. Alcohol intake is an example of a possible factor not included, just as it was excluded from our study.

There is growing evidence that smoking increases the visceral accumulation of fats, insulin resistance, and the frequency of metabolic syndrome and type 2 diabetes mellitus [6,19,20]. Smokers have a larger abdominal circumference, but smaller hip circumference [6,21]. Also, it is known that cessation of smoking is associated with an increase in BMI due to the increase in body fat, especially abdominal fat [22,23].

Kamyo et al. [24] discussed the lack of consensus on the role of smoking in obesity. They found that smoking was an independent risk factor for visceral accumulation of fats, and speculated that the use of tobacco products could cause insulin resistance and hyperinsulinemia and the central accumulation of fat. Volunteers with the highest smoking rates had an OR of 1.7 for hyperglycemia in relation to non-smokers, showing the effect of smoking load on glucose and lipid metabolism in the sample of subjects.

In another prospective study, LaRowe et al. [25] reported that treatment-seeking smokers have a higher prevalence of obesity compared to current smokers in the general population, suggesting that the treatment seekers may have a different health profile.

The case-control design of this study and the size of the sample (n = 1022), with complete data, allowed us to carry out a differentiated statistical analysis. The results regarding the frequency of smoking in the control group matched the inquiries of the local populations, detecting approximately 25% of smokers. This shows that the control group was very representative of the population of the city of Porto Alegre [26]. Buchwald et al. [27] reported a similar percentage of smokers or former smokers (24.2% of 1881 patients in a total of 19,388 individuals).

There were limitations to the current study. Since this study involved a survey of information from a databank, it was not possible to evaluate the associated frequency with other compulsions and the waist/hip ratio or even alcohol intake. Because our subjects were self-selected as a group of subjects seeking medical attention, our results cannot be applied to other subjects studied in different designs. Another point is that the education level of our patients and controls is higher when compared to the Brazilian general population. We realize that our findings must be confirmed by studies specifically addressing these and other points.

Considering that smoking is an important risk factor for adverse outcomes in bariatric surgery [28], we believe that additional studies are necessary in the field (smoking and obesity) in order to familiarize the health professionals with the special needs of this group of individuals.

## Conclusions

In this study it was possible to evaluate the relationships between smoking and body weight/BMI in various categories or as a continuous variable in a center specialized in obesity treatment. Previous knowledge of the inverse association between BMI and smoking, already well-defined, was confirmed in individuals with normal weight. This study also confirms that in overweight and obese patients, the tendency was distinct; as BMI increased, the frequency of smokers increased linearly. What this study brings to light is that such findings are also repeated in morbidly obese individuals. The external validity of these findings must be confirmed by other studies because our data included a very specific group of subjects.

## Competing interests

The authors declare that they have no competing interests.

## Funding sources

This study had no funding source.

## Authors' contributions

All authors contributed to and have approved the final version of the manuscript.

RC conducted the study and the literature search.

CCM wrote the protocol and conducted the statistical analyses.

JMC wrote the protocol and prepared the manuscript from the first draft to the final version of the manuscript.

## Pre-publication history

The pre-publication history for this paper can be accessed here:

http://www.biomedcentral.com/1471-2466/10/61/prepub
